# Alterations in the Contra lateral Ear in Chronic Otitis Media

**Published:** 2013

**Authors:** Mohammad Ali Damghani, Ali Barazin

**Affiliations:** 1 *Department of Otorhinolaryngology, Kerman University of Medical Sciences, Kerman, Iran.*

**Keywords:** Hearing impairment, Middle ear infection, Tympanometry

## Abstract

**Introduction:**

Chronic otitis media (COM), a persistent and durable inflammation and infection of the middle ear, is a common disorder. Alterations in the contralateral ear in sufferers have been observed in recent years. Because only a few studies have been reported in this area, we performed this study in order to assess alterations in the contralateral ear of patients with COM.

**Materials and Methods:**

Cross-sectional and descriptive methods were used in 100 patients with COM who were selected for surgical treatment and admitted to hospital. An information form was completed for all patients including demographic data, medical history of otoscopy and paraclinical examinations such as pure tone audiometry (PTA), tympanometry, Schuller radiography, and high resolution computed tomography (HRCT). All data were processed using SPSS (version 18) software and descriptive statistical tests.

**Results:**

According to otoscopy, PTA, tympanometry and graphical analysis, 60% of patients experienced disorders of the contralateral ear. Otoscopy analysis showed 54% of patients had a disorder of the contralateral ear, with the most common disorder being perforation of the ear drum. PTA showed a 48% incidence of contralateral ear problems (85% conductive hearing impairment; 12.5% sensorineural hearing impairment; 1.2% mixed). A total of 73.2% of patients with conductive hearing loss had a problem across all frequencies, while half of the patients with sensorineural hearing impairment had problems at frequencies greater than 1000 Hz. According to tympanometry, 38% of patients had problem in the contralateral ear. HRCT and Schuller graphical analyses indicated 31.5% and 36% occurrence of contralateral ear disorders, respectively.

**Conclusion:**

More than 50% of patients with COM in one ear have a chance of also presenting with the disease in the other ear. Outcomes of this study and previous studies have shown that COM should not be perceived as a disease limited to one ear, because in most cases the progress of the disease can affect both ears.

## Introduction

Otitis media, the most common disorder of the middle ear, is usually caused by infection and can be classified into acute otitis media (AOM), otitis media with effusion (OME), chronic suppurative otitis media, and chronic otitis media (COM) with cholesteatoma (1). COM is an inflammation and infection of the middle ear which is persistent and durable. The prevalence of COM varies around the world, affecting 30% of North America’s Eskimos, 4–6% of African populations and less than 1% of individuals in the United States and United Kingdom (2,3). Poverty, crowded living conditions due to large families, poor sanitation, and lack of personal and environmental hygiene are some of the main factors underlying the prevalence of COM (4). 

As the main cause of COM is a malfunction of the Eustachean tube, it is probable that a patient with COM will have a disorder of the contralateral ear (1). One of the most thorough studies in this area was performed in Brazil and indicated that 75% of COM patients also have some alteration in the contralateral ear. The most common finding was retraction, followed by perforation of the ear drum, cholesteatoma, and plaque sclerosis (5). Because of the shortage of literature on this subject, we performed the present study in order to describe alterations of the contralateral ear in patients with COM who were admitted to the Shafa Hospital of Kerman University between the years 2009 and 2010.

## Materials and Methods

This study was undertaken using a cross-sectional and descriptive methodology. Patients over 15 years of age who were admitted to the ear, nose, and throat (ENT) unit of Shafa Hospital between 2009 and 2010 with a diagnosis of COM were entered into this assessment. An information form was completed for all patients, requiring demographic data including age, gender, occupation, and level of education. Causative factors, chief complaint, and associated symptoms (including putrid or regular otorrhea, hearing impairment, dizziness, tinnitus, earache, or signs of fistula [dizziness after inducing pressure into external ear canal]) were also recorded. Next, an otoscopy was performed with evidence of perforation of the ear drum, plaque sclerosis, neotympanum, retraction of tympanum, or existence of otorrhea (regular or putrid) recorded on the form. Pure tone audiometry (PTA) was performed, as well as an assessment of the ear drum by tympanometry, and imaging of the contralateral ear by Schuller radiography and high resolution computed tomography (HRCT). Finally data were analyzed using SPSS (version 18) statistical software and descriptive statistical tests.

## Results

Among 100 patients with COM, 47% were male and 53% were female, with average age of 23.03±12.9 years (range, 15–63). The average duration of disease was 11.87±10.9 years (range, 1–15). Most patients (36.1%) were housewives and approximately half (49.5%) were educated to a level below high school diploma. Fifty-three percent of patients had COM in the left ear, with the causative factor in 47.5% of patients being AOM. The chief complaint in 56% of patients was otorrhea, followed by hearing impairment (37%).

According to the results of otoscopy, PTA, tympanometry and imaging, 60% of patients had a disorder of the contralateral ear, while otoscopy showed 54% of patients had a problem in the contralateral ear. It should be noted that some patients had overlapping symptoms. The most common symptoms were perforation of the ear drum (59.2%) and plaque sclerosis (44.4%) (Table 1). The results of PTA indicated 48% hearing impairment in the contralateral ear (Table 2), with 73.2% of patients experiencing conductive hearing loss across all frequencies, 12.2% at frequencies of 250 and 500 Hz, 7.3% at frequencies of 250, 500 and 1000 Hz, and

7.3% at frequencies of more than 2000 Hz. Moreover, 50% of patients had sensorineural hearing impairment at frequencies of 1000, 2000 and more than 2000 Hz, 33.3% across all frequencies, and 16.7% at frequencies of greater than 2000 Hz.

According to the results of tympanometry, 38% of patients had a disorder in the contralateral ear (Table 3). 

In 75 patients, Schuller radiography was performed which indicated that 36% of patients had a disorder in the contralateral ear, of which the most common was a decline in mastoid air cells (Table 4). HRCT was performed in 38 patients and showed that 31.5% had a problem in the contralateral ear (Table 5). Among these, 58.3% revealed a decline in air mastoid cells, 16.7% had sclerosis and 25% exhibited erosion of the temporal bone. Thirteen patients underwent both HRCT and Schuller radiography. 

**Table 1:** Otoscopic results, number of cases.

**Tympanum** **perforation** **n (%)**	**Tympanosclerosis** **n (%)**	**Regular otorrhea** **n (%)**	**Tympanum** **retraction** **n (%)**	**Neotympan** **n (%))**	**Putrid** **otorrhea** **n (%)**
32 (59.2)	24 (44.4)	12 (22.2)	6 (11.1)	6 (11.1)	3 (5.5)

Some symptoms were overlapping in some patients

**Table 2:** PTA results.

Conductive	Sensoryneural	Mixed	Total
41) 85.4)	6 (12.5)	1 (2.1)	48) 100)

**Table 3:** Tympanometry results.

Type B	Type C	Total
34 (89.5)	4 (10.5)	38 (100)

**Table 4:** Schuller results.

Decline in air mastoid cells	Sclerosis	Bone defect	Total
16 (59.3)	9 (33.3)	2 (7.4)	27 (100)

**Table 5:** HRCT results.

Decline in air mastoid cells	Sclerosis	Bone defect	Total
7 (58.3)	2 (16.7)	3 (25)	12 (100)

## Discussion

Inflammation and infection in middle ear is known as COM, and is persistent and long-lasting, with varying prevalence around the world (2,3). Poverty, over-populated families, and poor environmental and personal sanitation are some of the main causes of the condition (4). Malfunction of the Eustachean tube of varying types plays an important role in this disease (1). According to the continuum theory, OM with effusion (OME) is recognized as an initial condition which, when unresolved, may progress to chronic transformation. Although only a small percentage of cases of OME will evolve to COM, considering that the presence of bilateral effusion is reported to be high (6), it might be expected that the prevalence of bilateral COM would be similarly prevalent. The major purpose of this study was therefore to investigate the contralateral ear in patients with COM. 

Limited data are currently available in the literature relating to the contralateral ear in patients with COM (7,8). Previous studies have predominantly focused on the condition of the contralateral ear via otoscopy, but in our study we used PTA, tympanometry, and radiography in addition to otoscopy for the evaluation of the contralateral ear in COM. One hundred patients (47 male and 53 female) were included in the study, with an average age of 23.03 years. In a previous study performed in Brazil, the average age was 26.3 years, and in two other Iranian studies it was 30 and 32 years, respectively (9,10). The average duration of disease in our study was 11.87 years, compared with 6.5 years as reported in an earlier study. (11). The main cause of the disease was AOM, and the chief complaint of patients was hearing impairment and purulent drainage of the ear, whereas in other studies these factors were disregarded. In this study, the incidence of problems in the opposite ear based on otoscopy, PTA, tympanometry and radiographic techniques was 60%. Furthermore, according to the results of otoscopy, ear-drum perforation and the existence of plaque sclerosis in the ear drum were the principal problems. In a study from 1984 in 73 patients with COM, 54.4% had a problem in the contralateral ear, with retraction being the most common finding (7). In another study from 1996 in 496 patients with COM, 63% had disorders in their contralateral ear, with retraction again the most common disorder followed by perforation of the tympanum (8). In a study in 500 patients in Brazil, 75.2% had a problem in the contralateral ear, in which retraction of the tympanum was the primary feature (5). In this study, PTA analysis showed that 48% of patients had hearing impairment in the contralateral ear, which was conductive in 85% of cases, sensorineural in 12.5% and a mixed hearing impairment in 1.2%. By means of tympanometry, 38% of patients were diagnosed with a problem in the contralateral ear, of which 89.5% was type B and 10.5% was type C. Assessment with imaging (Schuller radiography and HRCT) indicated 36% and 31.5% of disorders in the contralateral ear, respectively, of which a decline in the mastoid air cells was the most common disorder. Findings of PTA, tympanometry, and imaging were not considered in earlier studies, and we are therefore unable to compare our results with earlier findings in this regard.

## Conclusion

In more than 50% of patients with COM, diverse disorders in the contralateral ear may be present. The results of this study and previous studies show that we should not consider COM as a disease limited to one ear because in many cases the occurrence of this disease can affect both ears. This issue should always be clarified in patients in order to achieve effective therapeutic planning. Consequently, the contralateral ear should always be evaluated comprehensively in patients with unilateral COM to efficiently diagnose any alterations and, if necessary, provide timely therapeutic intervention.
